# Imaging of dopamine transporters in Parkinson disease: a meta‐analysis of ^18^F/^123^I‐FP‐CIT studies

**DOI:** 10.1002/acn3.51122

**Published:** 2020-08-14

**Authors:** Yanyan Kong, Chencheng Zhang, Kawai Liu, Aparna Wagle Shukla, Bomin Sun, Yihui Guan

**Affiliations:** ^1^ Department of Neurosurgery, Center for Functional Neurosurgery Ruijin Hospital Shanghai Jiao Tong University School of Medicine Shanghai 200025 China; ^2^ PET Center Huashan Hospital Fudan University Shanghai 200235 China; ^3^ Department of Mathematics The Shanghai SMIC Private School Shanghai 200000 China; ^4^ Department of Neurology and Fixel Center for Neurological Diseases and the Program for Movement Disorders and Neurorestoration University of Florida Gainesville FL 32611

## Abstract

**Objective:**

^18^F‐FP‐CIT and ^123^I‐FP‐CIT are widely used radiotracers in molecular imaging for Parkinson’s disease (PD) diagnosis. Compared with ^123^I‐FP‐CIT, ^18^F‐FP‐CIT has superior tracer kinetics. We aimed to conduct a meta‐analysis to assess the efficacy of using ^18^F‐FP‐CIT positron emission tomography (PET) and ^123^I‐FP‐CIT single‐photon emission computed tomography (SPECT) of dopamine transporters in patients with PD in order to provide evidence for clinical decision‐making.

**Methods:**

We searched the PubMed, Embase, Wanfang Data, and China National Knowledge Infrastructure databases to identify the relevant studies from the time of inception of the databases to 30 April 2020. We identified six PET studies, including 779 patients with PD and 124 healthy controls, which met the inclusion criteria. Twenty‐seven SPECT studies with 1244 PD patients and 859 controls were also included in this meta‐analysis.

**Results:**

Overall effect‐size analysis indicated that patients with PD showed significantly reduced ^18^F‐FP‐CIT uptake in three brain regions [caudate nucleus: standardized mean difference (SMD) = −1.71, *Z* = −3.31, *P* = 0.0009; anterior putamen: SMD = −3.71, *Z* = −6.26, *P* < 0.0001; and posterior putamen: SMD = −5.49, *Z* = −5.97, *P* < 0.0001]. Significant decreases of ^123^I‐FP‐CIT uptake were also observed in the caudate (SMD = −2.31, *Z* = −11.49, *P* < 0.0001) and putamen (SMD = −3.25, *Z* = −14.79, *P* < 0.0001).

**Interpretation:**

In conclusion, our findings indicate that both ^18^F‐FP‐CIT PET and ^123^I‐FP‐CIT SPECT imaging of dopamine transporters can provide viable biomarkers for early PD diagnosis.

## Introduction

Parkinson’s disease (PD) is the second most common neurodegenerative disorder.^1^ Its predominant pathological feature is the degeneration of dopaminergic neurons projecting from the substantia nigra pars compacta in the midbrain to the striatum (caudate and putamen nucleus). It clinically presents with motor impairment, including rigidity, bradykinesia, resting tremor, and postural imbalance.^2^, ^3^ However, early diagnosis of PD based on clinical signs and symptoms is difficult.^4^ Furthermore, PD diagnosis solely based on clinical assessment is not always accurate since the clinical symptoms of PD could be similar to those of other neurodegenerative diseases. Moreover, studies have reported that at least 15% of the patients diagnosed with PD do not meet the clinical diagnostic criteria, while 20–25% were diagnosed with another condition, including atypical parkinsonian syndromes or essential tremor.^5^, ^6^, ^7^, ^8^

Currently, apart from the clinical symptoms, some functional brain imaging modalities have been reported to increase the accuracy of PD diagnosis.^9^, ^10^ Dopaminergic dysfunction in patients with PD can be visualized through single‐photon emission computed tomography (SPECT) and positron emission tomography (PET) using various radiotracers. SPECT derivatives include ^123^I‐FP‐CIT (DatScan™), ^123^I‐beta‐CIT (DopaScan™), ^123^I‐altropane, and ^99m^Tc‐TRODAT, while PET tracers include ^11^C‐RTI‐32, ^11^C‐CFT, ^11^C‐methylphenidate, ^11^C‐nomifensine, ^11^C‐dihydrotetrabenazine, ^18^F‐DOPA, ^18^F‐FE‐PE2I, ^18^F‐CFT, and ^18^F‐FP‐CIT.^11^, ^12^, ^13^, ^14^, ^15^, ^16^, ^17^, ^18^

Compared with ^18^F‐FP‐CIT, ^123^I‐FP‐CIT has been shown to have a higher affinity for the presynaptic dopamine transporter and a longer half‐life (6–13 h); furthermore, it has been commercially available for auxiliary diagnosis of PD using SPECT.^9^, ^19^ However, considering their higher nonspecific background signals, SPECT radiotracers, including ^123^I‐FP‐CIT and ^123^I‐altropane, exhibit lower striatal/cerebellar uptake ratios.^16^ ^18^F‐DOPA, which is an effective substitute tracer for ^123^I‐FP‐CIT, has been reported to underestimate the degree of nigrostriatal impairment in SPECT imaging due to the upregulation of aromatic amino acid decarboxylase as a compensatory mechanism in the presynaptic dopaminergic nerve terminal.^6^

In contrast, ^18^F‐FP‐CIT has a shorter half‐life (20–110 min) and superior tracer kinetics compared to other SPECT tracers; furthermore, it is well set up for detecting presynaptic dopaminergic deficiency in various parkinsonian syndromes.^20^, ^21^ Previous studies have reported a significantly faster decrease in striatal ^18^F‐FP‐CIT uptake in patients with PD compared to that in controls. Moreover, there are differences in the rates of decrease among the striatal subregions, namely, the caudate nucleus, anterior putamen, and posterior putamen, which indicate that ^18^F‐FP‐CIT may be more effective for the diagnosis of PD.^22^, ^23^, ^24^, ^25^, ^26^

However, there is heterogeneity among the studies, such as differences in the sample sizes, study design, and participant characteristics, as well as inadequate comparability and evidentiary value of findings from striatal subregion analysis. To the best of our knowledge, a meta‐analysis of previous studies on ^18^F‐FP‐CIT PET/^123^I‐FP‐CIT SPECT imaging of dopamine transporters for the diagnosis of PD has not been conducted to date. Therefore, we aimed to collectively evaluate ^18^F‐FP‐CIT PET/^123^I‐FP‐CIT SPECT imaging findings of dopamine transporters in patients with PD, identify patterns of abnormalities, and propose evidence that can influence clinical decision‐making with respect to the diagnosis of PD.

## Materials and Methods

### Search strategy and study selection

We searched for relevant studies in the PubMed, Embase, Wanfang Data, and China National Knowledge Infrastructure databases from the time of inception of the databases to 30 April 2020. This search included both English and Chinese articles. We used the following key terms: “^18^F‐FP‐CIT,” “^123^I‐FP‐CIT,” “Paralysis Agitans,” “Parkinson’s disease,” “Idiopathic Parkinson disease,” “Parkinsonism,” “atypical Parkinsonism” “positron emission tomography,” and “single‐photon emission computed tomography.” The references of the eligible articles were manually screened to retrieve potentially relevant studies deemed suitable for analysis. The inclusion criteria for studies in the meta‐analysis were as follows: assessment of ^18^F‐FP‐CIT/^123^I‐FP‐CIT uptake in the caudate nucleus or putamen (anterior and posterior putamen) while performing PET/SPECT and reporting of the corresponding outcomes [standardized uptake value ratio (SUVR)] in PD patients and healthy control subjects. We included cross‐sectional studies, case‐control studies, cohort studies, and randomized controlled trials. We excluded case reports, letters to the editor not subjected to peer review, conference abstracts, as well as studies without well‐defined cohorts of patients with PD, those without controls or consecutive patients seen in a unit, and those without quantitative outcomes.

Two independent investigators (Yanyan Kong and Chencheng Zhang) conducted the entire literature search and study selection process. In case of disagreements, the reviewers discussed their viewpoints, and a third investigator (Bomin Sun) was consulted if no consensus was reached. For studies that recruited participants within the same time period or from the same hospital, we only included the study with the largest sample size or the most pertinent outcomes to avoid duplication.

### Data extraction and statistical analysis

R software (version 3.6.1; The R Foundation, Vienna, Austria) and Statistical Analysis System (version 9.3; SAS Institute, Cary, NC) were used for statistical analysis. Several outcome measurements can be used in PET/SPECT studies on dopamine transporter binding in PD. Among them, regional SUVR or standardized uptake values measured using subject‐specific FreeSurfer‐generated volume of interest are considered the gold standard.^22^ Two investigators (Yanyan Kong and Chencheng Zhang) independently extracted data from each study. The following variables were retrieved: name of the first author, year of publication, sample size, disease duration, age of the patients with PD and controls, and estimation of effects (means and standard deviations for the decrease in SUVR in the PD group compared with the healthy control group).

The standardized mean difference (SMD) and standard error (SE) of the SMD for the outcomes in the PD and control groups were calculated separately for the reported brain regions in each study. SMDs were used because the main outcomes of interest in PET/SPECT studies of dopamine transporter binding can be assessed by several possible outcome measurements.^27^. Random‐effects meta‐analysis using the DerSimonian and Laird method was considered due to high heterogeneity across the studies. *I* ^2^ statistics were used to assess the heterogeneity among the included studies. *P* < 0.05 was considered to indicate significant heterogeneity in the *I* ^2^ statistics test. Additionally, *I* ^2^ values of 0–25%, >25–50%, >50–75%, and >75% indicated insignificant, low, moderate, and high heterogeneity, respectively.^28^ We had initially planned to assess the potential impact of moderator variables; however, the included studies did not provide adequate information for such examination. Given the small number of studies across all investigated domains, publication bias assessment using funnel plots was not suitable.^28^, ^29^, ^30^, ^31^, ^32^ Therefore, publication bias was assessed using Rosenberg’s Fail‐safe N approach.^33^ A fail‐safe number represents the number of nonsignificant and unpublished (or missing) studies that must be included in the meta‐analysis to render an observed statistically significant result nonsignificant.^33^, ^34^ We calculated the fail‐safe N for each significant outcome, and results at *P* < 0.05 were considered statistically significant.

## Results

### Study selection and characteristics

**Figure 1:**
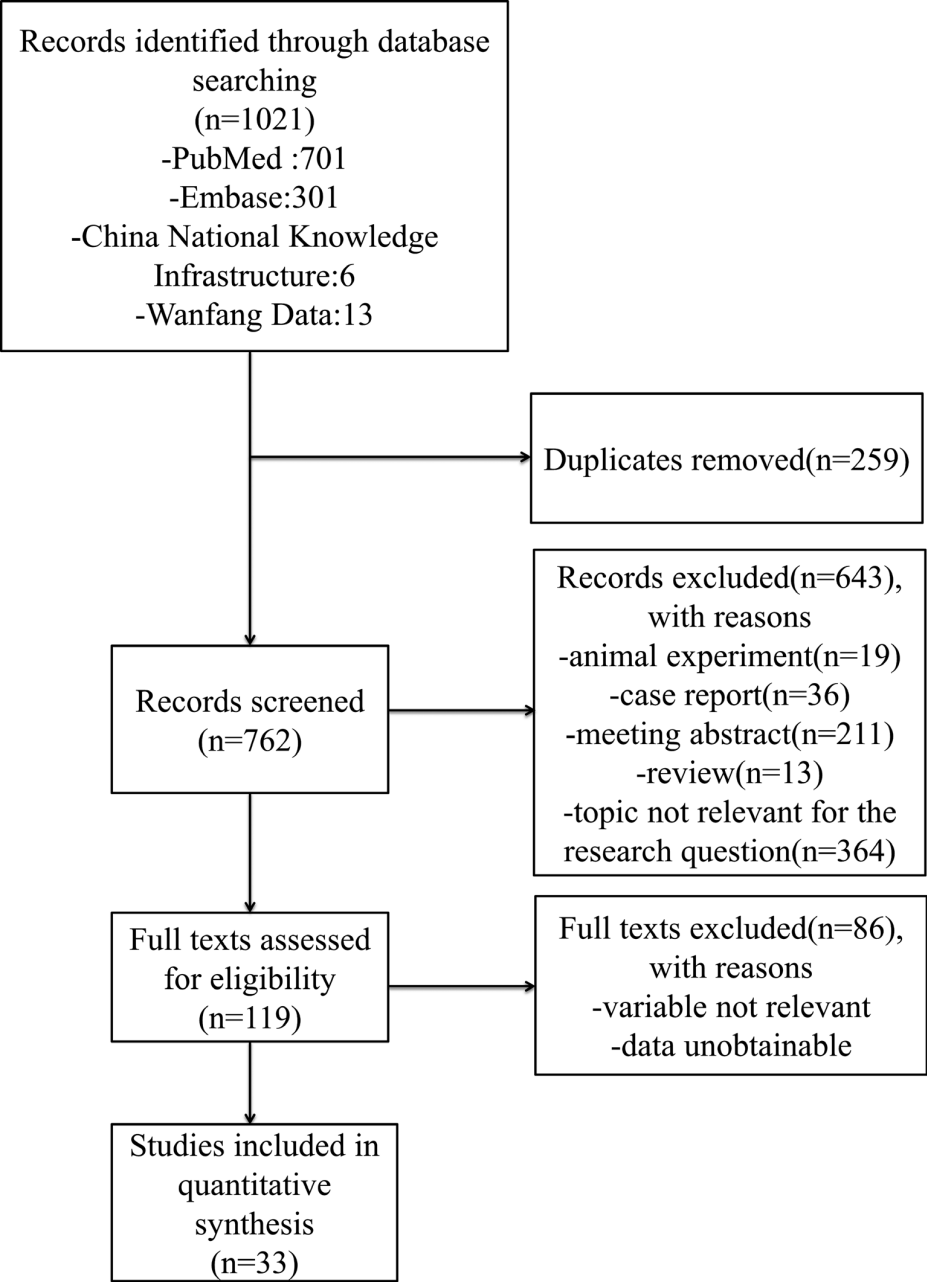
Search results and flow chart for the inclusion of studies in the meta‐analysis.

The initial literature search yielded 1021 studies, from which we eliminated 259 duplicated studies and 643 articles that did not meet the inclusion criteria. The excluded articles included animal experiments (19), case reports (36), meeting abstracts (211), reviews (13), and topics not pertinent to the research question (303 were not ^18^F‐FP‐CIT PET/^123^I‐FP‐CIT SPECT studies, 37 were not PD studies, and 24 lacked quantitative outcomes). The preliminarily selected 119 full‐text articles were reviewed for eligibility, after which 86 were excluded for reporting irrelevant variables and unobtainable data. We included the remaining 33 articles (six articles reported PET studies involving 779 patients with PD and 124 controls, and 27 reported SPECT studies involving 1244 PD patients and 859 controls) in this meta‐analysis. These studies comprised cross‐sectional surveys (10 studies), case‐control studies (19 studies), and cohort studies (four studies).^17^, ^22^, ^23^, ^24^, ^25^, ^26^, ^35^, ^36^, ^37^, ^38^, ^39^, ^40^, ^41^, ^42^, ^43^, ^44^, ^45^, ^46^, ^47^, ^48^, ^49^, ^50^, ^51^, ^52^, ^53^, ^54^, ^55^, ^56^, ^57^, ^58^, ^59^, ^60^, ^61^ Figure 1 illustrates the protocol for the literature search and study selection process. Table 1 summarizes the characteristics of the individual studies, including the sample sizes and participant characteristics. These studies were published from 1998 to 2019. Furthermore, except for one study on the caudate that did not report significant between‐group differences, the remaining studies reported decreased ^18^F‐FP‐CIT uptake.

**Table 1:** Characteristics of the included studies.

Study	PD (n)	HC (n)	PD age (years)	HC age (years)	PD sex (M/F)	HC sex (M/F)	Disease duration (years)	Tracer
Wang 2003	31	4	57 ± 8	59 ± 8	–	–	–	^18^F‐FP‐CIT
Wang 2006	41	12	58 ± 8	59 ± 7	–	–	4.3 ± 3.4	^18^F‐FP‐CIT
Song 2013	24	18	70 ± 8	70 ± 10	18/6	11/7	1.9 ± 0.7	^18^F‐FP‐CIT
Kim 2015	56	71	61 ± 12	60 ± 10	31/25	39/32	2.7 ± 2.8	^18^F‐FP‐CIT
Sung 2017	35	9	55 ± 12	70 ± 8	14/21	7/2	2.5 ± 2.8	^18^F‐FP‐CIT
Yang 2017	592	10	70 ± 6	70 ± 8	40/502	2/8	6.0 ± 4.7	^18^F‐FP‐CIT
Tissingh 1998	21	14	56 ± 10	54 ± 15	15/6	7/7	2.3 ± 1.3	^123^I‐FP‐CIT
Booij 1999	19	10	57	53	18/1	1/9	–	^123^I‐FP‐CIT
Booij 2001	32	36	53 ± 11	53 ± 18	24/8	16/20	2.0 ± 1.3	^123^I‐FP‐CIT
Ceravolo 2004	20	8	67 ± 8	66 ± 9	13/7	5/3	4.3 ± 1.9	^123^I‐FP‐CIT
O’Brien 2004	38	33	76 ± 5	75 ± 6	28/10	17/16	5.3 ± 7.3	^123^I‐FP‐CIT
Tsuchida 2004	6	10	58 ± 12	36 ± 16	4/2	6/4	8.0 ± 4.7	^123^I‐FP‐CIT
van Laere 2004	39	10	62 ± 11	60 ± 16	23/16	6/4	2.1 ± 1.3	^123^I‐FP‐CIT
Varrone 2004	9	6	44 ± 3	51 ± 13	4/5	5/1	–	^123^I‐FP‐CIT
Colloby 2005	20	22	74 ± 5	72 ± 4	14/6	13/9	4.5 ± 4.6	^123^I‐FP‐CIT
Filippi 2005	29	18	61 ± 11	62 ± 8	16/13	9/9	2.2 ± 1.5	^123^I‐FP‐CIT
Filippi 2006	21	20	64 ± 8	64 ± 7	12/9	9/11	2.7 ± 1.9	^123^I‐FP‐CIT
Canesi 2007	38	14	66 ± 8	–	25/13	–	9.6 ± 4.0	^123^I‐FP‐CIT
Isaias 2007	20	31	60 ± 11	–	11/9	–	1.5 ± 1.0	^123^I‐FP‐CIT
Isaias 2008	47	31	62 ± 10	64 ± 10	28/19	13/18	3.0 ± 1.0	^123^I‐FP‐CIT
Varrone 2008	24	6	46 ± 10	51 ± 13	16/8	5/1	–	^123^I‐FP‐CIT
Isaias 2010	13	23	63 ± 9	71 ± 9	7/6	10/13	5.0 ± 2.8	^123^I‐FP‐CIT
Kim 2010	14	12	67 ± 4	63 ± 6	11/3	8/4	–	^123^I‐FP‐CIT
Cilia 2011	37	32	70 ± 5	70 ± 6	18/19	15/17	4.4 ± 2.9	^123^I‐FP‐CIT
Djaldetti 2011	15	11	68 ± 9	61 ± 12	7/8	–	20.6 ± 6.3	^123^I‐FP‐CIT
Isaias 2011	11	10	52	52	5/6	3/7	–	^123^I‐FP‐CIT
Di Giuda 2012	21	17	60 ± 13	55 ± 14	14/7	7/10	4.0 ± 2.1	^123^I‐FP‐CIT
Siepel 2014	339	158	61 ± 10	60 ± 11	219/120	98/60	–	^123^I‐FP‐CIT
Roussakis 2016	28	12	69 ± 5	61 ± 9	19/9	7/5	9.8 ± 5.0	^123^I‐FP‐CIT
Jakobson 2018	22	28	69 ± 8	70 ± 4	–	–	1.1 ± 0.8	^123^I‐FP‐CIT
Nicastro 2019	280	208	70 ± 11	70 ± 11	148/132	83/125	1.3 ± 0.8	^123^I‐FP‐CIT
Pilotto 2019	56	54	65 ± 10	61 ± 13	40/16	35/19	2.2 ± 2.1	^123^I‐FP‐CIT
Wilson 2019	25	25	54 ± 8	46 ± 8	11/14	10/15	9.6 ± 3.9	^123^I‐FP‐CIT

Values are means ± standard deviation or *n*.

PD, Parkinson’s disease; HC, healthy controls; M, male; F, female.

### Estimates of effect size

**Figure 2:**
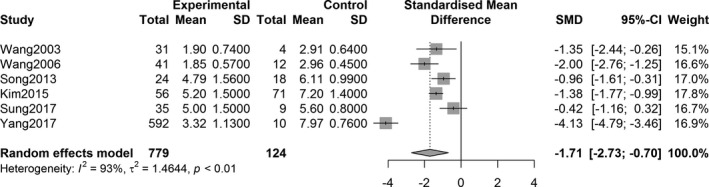
Forest plot for ^18^F‐FP‐CIT PET studies assessing the decrease in SUVR in the caudate.

**Figure 3:**
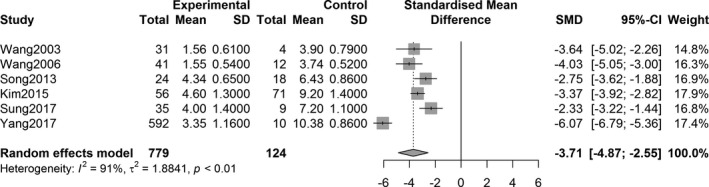
Forest plot for ^18^F‐FP‐CIT PET studies assessing the decrease in SUVR in the anterior putamen.

**Figure 4:**
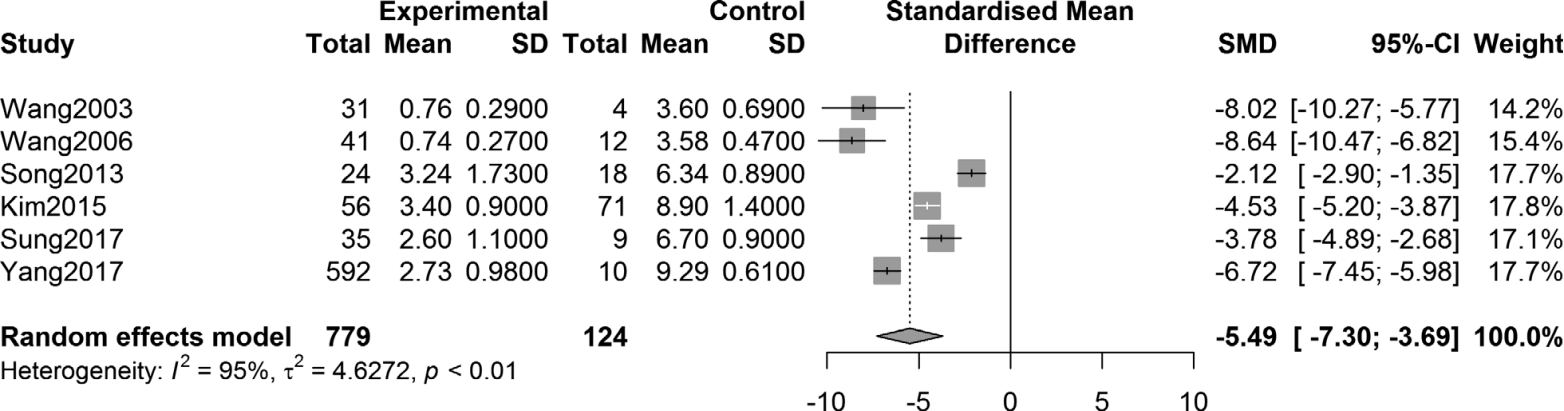
Forest plot for ^18^F‐FP‐CIT PET studies assessing the decrease in SUVR in the posterior putamen.

**Figure 5:**
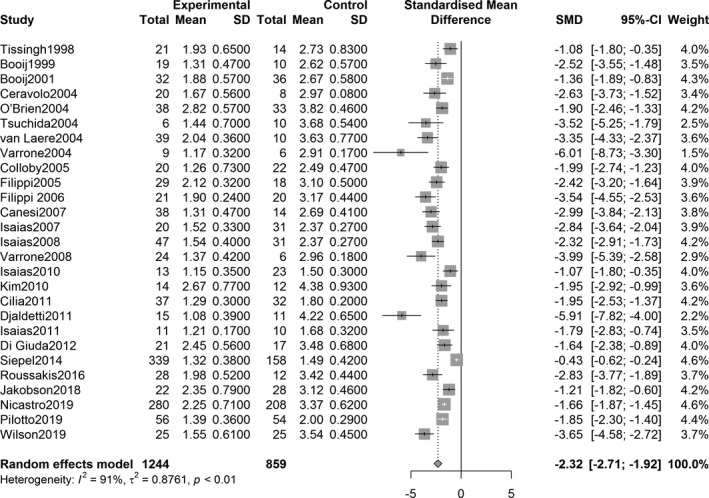
Forest plot for ^123^I‐FP‐CIT SPECT studies assessing the decrease in SUVR in the caudate.

**Figure 6:**
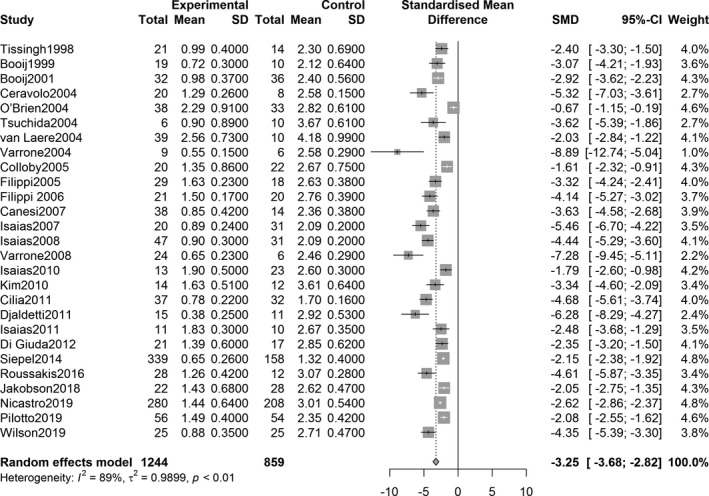
Forest plot for ^123^I‐FP‐CIT SPECT studies assessing the decrease in SUVR in the putamen. Std., standard; CI, confidence interval; SUVR, standardized uptake value ratio.

The overall effect analysis of PET studies indicated a statistically significant decrease in the SUVR in the three striatum regions between PD patients and controls, as follows: caudate region, SMD = −1.71, 95% confidence interval (CI) [−2.73, −0.70], *Z* = −3.31, *P* = 0.0009; anterior putamen, SMD = −3.71, 95% CI [−4.87, −2.55], *Z* = −6.26, *P* < 0.0001; and posterior putamen, SMD = −5.49, 95% CI [−7.30, −3.69], *Z* = −5.97, *P* < 0.0001. Figures 2, 3, 4 show the forest plots for the estimates of the effect size. Compared with the controls, patients with PD showed significant decrease in the uptake level in both caudate and putamen in the 27 SPECT studies (caudate, SMD = −2.31, 95% CI [−2.71, −1.92], *Z* = −11.49, *P* < 0.0001; putamen, SMD = −3.25, 95% CI [−3.68, −2.82], *Z* = −14.79, *P* < 0.0001). Figures 5 and 6 show the forest plots for overall effect sizes of the SPECT studies.

### PET studies on atypical parkinsonism

Four studies^21^, ^62^, ^63^, ^64^ reported the application of ^18^F‐FP‐CIT PET in atypical Parkinsonism. They suggested that ^18^F‐FP‐CIT PETs could be useful for the differential diagnosis of atypical Parkinson.

### Heterogeneity

With respect to the assessment of ^18^F‐FP‐CIT uptake, there was heterogeneity in the three striatal regions of interest (caudate: *I* ^2^ = 90%, *P* < 0.0001; anterior putamen: *I* ^2^ = 91%, *P* < 0.0001; and posterior putamen: *I* ^2^ = 95%, *P* < 0.0001). Statistical significance was observed in all heterogeneity tests (Figs. 2, 3, 4). The analysis also showed heterogeneity in the ^123^I‐FP‐CIT studies included (caudate, *I* ^2^ = 89%, *P* < 0.0001; putamen, *I* ^2^ = 91%, *P* < 0.0001) (Figs. 5, 6).

### Publication bias

The calculation of Rosenberg’s fail‐safe numbers indicated that a large number of unpublished and nonsignificant studies (effect sizes of zero) would be needed to achieve a *P*‐value >0.05 for the effect size of meta‐analyses of both PET and SPECT studies. A total of 418, 1966, and 4172 studies would be required to achieve *P* > 0.05 in meta‐analyses of ^18^F‐FP‐CIT PET imaging of the caudate, anterior putamen, and posterior putamen regions, respectively. Furthermore, for ^123^I‐FP‐CIT SPECT studies, Rosenberg’s fail‐safe numbers were 18556 and 41260 for the pooled analysis of the caudate and putamen.

## Discussion

### Summary of the findings

It is well‐known that findings from studies with small sample sizes are less powerful.^65^ Consequently, meta‐analyses are considered to be powerful and widely used tools that compile results from different studies to draw more meaningful conclusions than those of individual reports.^27^, ^66^ In the meta‐analysis of PET studies, the mean number of participants in the PD and control groups in each included study was 129 and 21, respectively. This disparity in the sample sizes mainly resulted from the imbalance in the number of participants recruited in Yang’s study (592 patients with PD and 10 controls). As shown in Table 1, the number of participants in the other five included studies met the sample size requirements for a typical molecular imaging study.^67^, ^68^, ^69^, ^70^, ^71^, ^72^, ^73^, ^74^, ^75^ With respect to individual studies, all included studies, except one (findings in the caudate nucleus in Sung’s study^24^), reported a significant decrease in the SUVR for ^18^F‐FP‐CIT uptake in the three striatum regions (caudate nucleus, anterior putamen, and posterior putamen) in patients with PD compared to that in the controls. Moreover, the overall effect size indicated significantly decreased ^18^F‐FP‐CIT uptake in the targeted cerebral regions. In addition, we performed a meta‐analysis of ^123^I‐FP‐CIT SPECT imaging studies. A total of 27 studies with 1244 PD patients and 859 healthy control subjects were included. The studies showed that patients with PD exhibited a significant decrease in uptake level in both the caudate and putamen compared to controls.

### Quality of meta‐analysis

*I* ^2^ statistical analysis was used to assess heterogeneity among the included studies. We found high heterogeneity in the results for the three investigated cerebral regions with *I* ^2^ values ranging from 90% to 95%. Moreover, we conducted a statistical analysis using a random effects model. The heterogeneity could be attributed to differences in the study designs, sample sizes, study locations, characteristics of the study populations, the severity of PD at the time of imaging, disease duration, category of complications, and correction for relevant factors. There are some limitations to this meta‐analysis. In the course of designing the study, we attempted to evaluate the potential impact of moderator variables using meta‐regression analysis; however, the included studies did not provide adequate information. Notably, Yang’s study enrolled 592 patients with PD and only 10 controls; therefore, we also performed a meta‐analysis after excluding this study. This led to a decrease in the *I* ^2^ values from 90% to 59%, 91% to 50%, and 95% to 94% in the caudate nucleus, anterior putamen, and posterior putamen, respectively. This indicated that allocation of participants to the PD and control groups in Yang’s study may be a potential source of bias. In this meta‐analysis, the number of studies included across all investigated domains was too small to assess publication bias using funnel plots; this may have reduced the statistical power of this study.^28^, ^29^, ^30^, ^31^, ^32^ Consequently, we calculated Rosenberg’s fail‐safe numbers, which were quite high as follows: N_C_ = 418, N_AP_ = 1966, and N_PP_ = 4172 (C: caudate, AP: anterior putamen, PP: posterior putamen). It has been suggested that possible publication bias could be safely ignored when the fail‐safe numbers are high.^33^, ^34^ Considering the limitations discussed above, the results of this analysis may have limited reference value and the observed correlations should be interpreted with caution.

### Clinical implications

Despite the limitations of this meta‐analysis, the overall effects showed significantly reduced ^18^F‐FP‐CIT/^123^I‐FP‐CIT uptake in patients with PD compared to that in the controls in cerebral regions of interest, which was consistently noted in the original studies included in this analysis. Therefore, ^18^F‐FP‐CIT/^123^I‐FP‐CIT are efficient tracers for primary PD diagnosis. Moreover, the six PET studies included reported that ^18^F‐FP‐CIT was effective not only in differentiating patients with PD from the healthy controls but also in determining the severity of PD.^22^, ^24^, ^25^, ^26^, ^35^ The progression rates differed significantly according to the various striatal subregions.^24^ In PET studies, the posterior putamen is the most severely affected region in the striatum while the caudate nucleus is relatively spared.^76^, ^77^, ^78^ However, data from some of these studies were not appropriate for assessment in this meta‐analysis. Nevertheless, we believe that our findings are robust, given the number of studies included. Well‐designed longitudinal studies are warranted to address issues regarding the detection of PD progression through ^18^F‐FP‐CIT PET/^123^I‐FP‐CIT SPECT imaging. Furthermore, the region of interest approach is a semi‐quantitative analysis method; more advanced neuroimaging techniques that can improve the diagnostic accuracy for PD or the differentiation of atypical Parkinsonism from PD are needed. Interestingly, studies on statistical parametric mapping, source‐based morphometry, and dual‐time ^18^F‐FP‐CIT reported promising findings with respect to the diagnosis of PD.^25^, ^79^, ^80^

In conclusion, our data indicate that ^18^F‐FP‐CIT PET and ^123^I‐FP‐CIT SPECT imaging of dopamine transporters is helpful in the diagnosis of PD. We propose that the application of PET or SPECT imaging modalities in combination with advanced approaches would be beneficial.

## Conflict of Interest

No potential conflict of interest relevant to this article exist.

Aparna Wagle Shukla –Reports grants from the NIH and has received grant support from Benign Essential Blepharospasm Research foundation, Dystonia Coalition, Dystonia Medical Research foundation, National Organization for Rare Disorders and grant support from NIH (KL2 and K23 NS092957‐01A1). Reports receiving honoraria from Acadia, Cavion, Elsevier, and MJFF in the past. Participates as a co‐I for several NIH, foundation, and industry sponsored trials over the years but has not received honoraria.
